# Low-Temperature Hydrothermal Synthesis of Rare-Earth-Doped Sc_2_Mo_3_O_12_ Crystals for Thermally Enhanced Upconversion Luminescence and Optical Temperature Sensing

**DOI:** 10.3390/ma19214628

**Published:** 2026-10-31

**Authors:** Yi Zuo, Yanlong Cao, Yonghong Yang, Juan Cai, Han Zhang, Junwei Hou

**Affiliations:** 1 Xinjiang Oilfield Company, Karamay 834000, China; 2 State Key Laboratory of Heavy Oil Processing, China University of Petroleum—Beijing at Karamay, Karamay 834000, China

**Keywords:** negative thermal expansion, one-pot hydrothermal reaction, scandium molybdate, upconversion luminescence

## Abstract

**Highlights:**

- A low-temperature hydrothermal method was developed to synthesize phase-pure orthorhombic Sc_2_Mo_3_O_12_ crystals.
- Phase-pure Sc_2_Mo_3_O_12_ enabled efficient multicolor upconversion luminescence (UCL) under 980 nm excitation.
- Thermally enhanced upconversion emission and optical thermometric performance were achieved over the temperature range of 310–670 K, with a maximum relative sensitivity (Sr) of 0.0028 K^−1^.

**Abstract:**

Thermal quenching severely limits the application of upconversion phosphors in high-temperature environments. Negative thermal expansion (NTE) materials have attracted considerable attention as promising hosts for enhancing high-temperature luminescence, whereas low-temperature synthesis of Sc_2_Mo_3_O_12_ remains rarely reported. In this work, phase-pure orthorhombic Sc_2_Mo_3_O_12_ crystals were synthesized via a low-temperature hydrothermal method. The effects of precursor molar ratio, reaction temperature, reaction time, and surfactant on phase formation were systematically investigated. Under the optimized synthesis conditions, efficient multicolor upconversion luminescence (UCL) was achieved under 980 nm excitation by employing Yb^3+^ as the sensitizer and Er^3+^, Tm^3+^, or Ho^3+^ as the activator ions. Only phase-pure Sc_2_Mo_3_O_12_ exhibited efficient UCL. Furthermore, the temperature- dependent luminescence properties were investigated over the range of 310–670 K, where the upconversion emission intensity continuously increased with temperature and a maximum relative sensitivity (Sr) of 0.0028 K^−1^ was obtained at 310 K based on the fluorescence intensity ratio (FIR) technique. These results demonstrate that phase-pure Sc_2_Mo_3_O_12_ prepared by a low-temperature hydrothermal method exhibits thermally enhanced UCL and promising optical thermometric performance, highlighting its potential as an NTE host for high-temperature optical temperature sensing.

## 1. Introduction

Upconverted luminescent materials are fast becoming a key instrument in the fields of bio-detection, optogenetics, advanced information anti-counterfeiting, optical temperature measurement, near-infrared imaging, and disease theranostics [1,2,3,4,5,6]. However, the non-radiative relaxation is a leading cause of the thermal quenching (TQ) problem severely limits its practical application and makes it difficult to carry out industrialized production [7,8,9,10].

Numerous studies have demonstrated that the NTE property of host materials can effectively enhance luminescence intensity through lattice contraction and distortion during heating, thereby mitigating thermal quenching at elevated temperatures [11,12,13,14,15,16,17,18,19,20]. This strategy has significantly broadened the application potential of upconversion luminescent materials in high-temperature environments [21]. Owing to its stable crystal structure and unique NTE behavior, Sc_2_Mo_3_O_12_ has been widely employed as a host material for rare-earth luminescent phosphors [22]. However, studies on the low-temperature synthesis of Sc_2_Mo_3_O_12_ phosphors remain scarce.

In this work, we aim to (i) develop a low-temperature hydrothermal route for phase-pure Sc_2_Mo_3_O_12_ crystals, (ii) investigate the influence of synthesis conditions on morphology and luminescence behavior, and (iii) evaluate their temperature-dependent UCL and optical thermometric performance. This work provides a feasible strategy for the low-temperature synthesis of NTE host materials and offers new insights into overcoming thermal quenching in high-temperature luminescent applications.

## 2. Materials and Methods

We used innovative techniques based on the recommendations of previous reports to synthesize Sc_2_Mo_3_O_12_ materials using a one-step hydrothermal method [23]. Sc(NO_3_)_3_·6H_2_O (99.99%), (NH_4_)_6_Mo_7_O_24_·4H_2_O (99.99%), Yb(NO_3_)_3_·5H_2_O (99.99%), ErH_12_N_3_O_15_ (99.99%) was obtained from damas-beta and without processing. The first step in the experiment was to configure all the materials in a certain concentration of solution using distilled water. Subsequently, the four solutions were added dropwise to 14 mL of deionized water and stirred in a PPL liner for 2 h. A white precipitate immediately formed. Sealed and lined with 50 mL stainless steel autoclave and heated for 2 h, heating temperature at 140 °C. The resulting samples were centrifuged, washed, dried at 85 °C and 800 °C for 2 h in an atmospheric environment using a muffle furnace. The synthesis method is shown in Figure 1.

The Phosphor phase composition and lattice parameters were carried out using an X-ray diffractometer (XRD, Ultima IV, Rigaku Corporation, Akishima, Tokyo, Japan). The morphologies and sizes of phosphors were observed by a scanning electron microscope (SEM, ZEISSSigma300, Oberkochen, Germany). UC spectra were recorded by a fluorescence spectrophotometer (F4700, Hitachi, Ltd., Tokyo, Japan) equipped with a 980 nm laser. The testing temperature was controlled by a thermal stage (THMS600, Linkam Scientific Instruments, Tadworth, UK).

In-situ variable-temperature X-ray diffraction (VT-XRD) measurements were carried out on a Bruker D8 Advance diffractometer with Cu Kα radiation (λ = 1.5406 Å) equipped with an Anton Paar HTK1200N high-temperature heating stage. The whole measurement was conducted under a continuous flowing high-purity nitrogen atmosphere to avoid oxidation, hydrolysis and surface contamination of the sample at high temperatures. Diffraction patterns were recorded from 140 °C to 200 °C with a temperature interval of 20 °C. At each target temperature, the sample was held for 8 min to achieve sufficient thermal equilibrium before data collection.

## 3. Results and Discussion

### 3.1. XRD Composition Analysis

After the completion of the reaction at 140 °C for 2 h according to different molar ratios of Sc:Mo, no crystals of the pure phase were prepared. From Figure 2a, we can see that there is a stray peak at 11.5°, and we guess that it may be the raw material that has not finished the reaction. Then, change the ratio of raw materials, and increase the amount of mo, still at 140 °C reaction 2 h, Figure 2b shows that the heterogeneous peaks are still present. Figure 2c illustrates the XRD patterns of the samples synthesized at Sc:Mo = 2:3 for 2 h in the solution with 140 °C, 160 °C, 180 °C and 200 °C. As shown in Figure 2c, it can be seen that the diffraction intensity is increasing with increasing temperature, but the spurious peaks are still present. Figure 2d illustrates the XRD patterns of the samples synthesized at the same molar ratio for 140 °C in the solution with 2 h, 6 h, 12 h and 24 h. As can be seen from Figure 2d, the diffraction intensity did not change with time and spurious peaks remained.

Phase-pure Sc_2_Mo_3_O_12_ was finally obtained after painstaking efforts. Crystals with different conditions were prepared according to Sc:Mo = 1:3. Figure 2e shows the XRD pattern of Sc_2_Mo_3_O_12_ particles at different temperatures for 2 h. The XRD pattern is in excellent agreement with the diffraction peaks of Sc_2_Mo_3_O_12_ (JCPDF No. 21-1329), demonstrating that Sc_2_Mo_3_O_12_ has been successfully synthesized via the hydrothermal method. As the temperature increases, the intensity of the diffraction peaks is increasing, and the location of the peaks does not change much. This suggests that increasing the temperature favours the growth of crystals. Figure 2f presents the XRD patterns of Sc_2_Mo_3_O_12_ synthesized by hydrothermal reaction at 140 °C for different reaction times. It can be seen that the intensity of the diffraction peaks increases with the extension of time, reaching the highest at 12 h, and the intensity of the diffraction peak decreases at 24 h, without much change in the location of the peak.

### 3.2. SEM Microscopic Morphology Analysis

Since crystal morphology and surface defects strongly influence luminescence efficiency, surfactant-assisted morphology regulation was further investigated. We used different surfactant assisted hydrothermal methods for surface modification and also performed SEM analysis to determine the microscopic morphology of the Sc_2_Mo_3_O_12_ crystals. Figure 3 highlights the differences in morphology after modification with EG, CATB and PVP. All reactions were carried out at 220 °C with 0.25 g urea for 24 h. Figure 3a shows the results of the surface morphology after glycol modification, which forms many rod-like and block-like regular structures. Ethylene glycol is miscible with water and can act as a co-solvent and coordinating agent during hydrothermal synthesis, thereby affecting crystal nucleation, growth, and morphology [24]. Figure 3b demonstrates the morphology of the rectangular lamellae formed after modification with CATB. A large number of catb molecules are adsorbed on the crystal surface, lowering the surface energy and directing crystal growth. From the data in Figure 3c, it is apparent that regular pie structures can be synthesized using PVP. PVP, as a water-soluble polymer compound, modifies the surface of nanoparticles to form a stable dispersion system, preventing aggregation and precipitation of nanoparticles [25]. Figure 3d,e can support this idea. We have successfully synthesized morphologically regular nano single crystals as can be seen in Figure 3f. Appendix A shows the XRD results of Sc_2_Mo_3_O_12_ materials modified with PVP, EG and CTAB, respectively. It can be observed that the diffraction peak intensity decreases slightly, while the peak positions remain almost unchanged. This confirms that the surfactants only regulate the crystal morphology without altering the crystal phase.

### 3.3. Up-Conversion and Down-Conversion Spectra

Yb^3+^ ions were introduced as sensitizers due to their efficient 980 nm absorption, while Er^3+^, Tm^3+^ and Ho^3+^ ions served as activators for generating multicolor emission.Figure 4a shows the UC emission spectra of the Sc_2_Mo_3_O_12_:Yb/Er (18%Yb, 1%Er) crystals under 980 nm excitation at room temperature. The green emission peaks at 524 and 559 nm are attributed to ^2^H_11/2_ → ^4^ I_15/2_ and ^4^S_3/2_ → ^4^ I_15/2_ transitions of Er ^3+^ ions. Figure 4b [26] showsthe UC spectra of the Yb/Ho-codoped Sc_2_Mo_3_O_12_ crystals under 980 nm excitation. We can observe intense red emission at 554 nm and 664 nm which are assigned to ^5^F_4_, ^5^S_2_ → ^5^I_8_ and ^5^F_5_ → ^5^I_8_ transitions, respectively, of Ho^3+^ ion. Figure 4c [27] shows the emission of blue light is mainly due to the ^3^H_6_ → ^1^G_4_ of Tm^3+^ ion.

Sc_2_Mo_3_O_12_ crystals also have excellent down-conversion luminescence properties. We measured the down-conversion emission spectra of samples doped with Yb/Er, Yb/Tm, and Yb/Ho with different doping concentrations under 980 nm laser excitation at room temperature, as shown in Figure 4d–f. Figure 4d demonstrates that down-conversion luminescence spectra of Sc_2_Mo_3_O_12_: Yb/Er (Yb: 5%, 10%, 15%, Er: 2%; Er:0.5%, 1%, 2%, 4%, Yb: 18%) with different doping concentrations. The highest fluorescence intensity was detected when the sensitizer was doped at a concentration of 5%. The characteristic emission peaks at 1525 nm NIR-IIb were attributed to ^4^I_13/2_ → ^4^I_15/2_ levels of the Er^3+^ ion, respectively. Yb^3+^ ions can act as energy traps to capture more energy effectively increasing luminescence in the near-infrared second region [28]. Then, the spectra demonstrated by Figure 4e,f are relatively simple compared to the up conversion spectra. Only infrared emission is observed in 1000–1150 nm, and the emission peak can be attributed to ^2^F_5/2_ → ^2^F_7/2_ levels of Yb^3+^ ion. Sc_2_Mo_3_O_12_: Yb/Tm (Yb: 5%, 10%, 15%, Tm: 2%; Tm: 0.5%, 1%, 2%, 4%, Yb: 18%) Sc_2_Mo_3_O_12_: Yb/Ho (Yb: 5%, 10%, 15%, Ho: 2%; Ho: 0.5%, 1%, 2%, 4%, Yb: 18%).

An important aspect was observed in these experiments, in which the fluorescence intensity could only be detected with pure-phase and well-ordered scandium crystals as the host material. Good morphology and stable nuclei contribute to efficient up conversion fluorescence from nanocrystals Sc_2_Mo_3_O_12_:x%Yb/Ln (Er, Tm, Ho).

### 3.4. UCL Fluorescent Temperature Sensing

Conventional materials expand upon heating, which reduces the energy transfer efficiency and weakens luminescence. In contrast, negative thermal expansion materials contract with increasing temperature and exhibit enhanced luminescence, laying a foundation for their application in optical thermometry. Figure 5 presents the high-resolution XRD patterns of Sc_2_Mo_3_O_12_ measured from 140 °C to 200 °C. The (112) diffraction peak (the strongest diffraction peak of this material) shifts rightward by 0.1° from 23.22° to 23.32°. Calculations based on Bragg’s law demonstrate that the interplanar spacing decreases by 0.00162 nm, confirming the reduction of interplanar spacing as temperature rises. Figure 6a shows the evolution of UCL spectra of Sc_2_Mo_3_O_12_:Yb/Er (18% Yb, 2% Er) under 980 nm excitation when the temperature increases from 310 K to 670 K. It can be observed that the luminescence intensity rises with increasing temperature without luminescence quenching.

Figure 6a shows the UCL spectra of Sc_2_Mo_3_O_12_:Yb/Er(18%Yb, 2%Er) in 310–670K, It can be seen that the fluorescence intensity increases with increasing temperature, and does not show decay. The Yb^3+^ and Er^3+^ thermal enhancement can be further used to achieve fluorescence intensity ratio thermometry. The relative intensity ratio fir of the radiative jumps of the two thermally coupled energy levels can be expressed as Equation (1).

(1) $$\mathrm{FIR}=C\exp(\frac{-∆E}{K_{B}T})$$

where C is a constant, ΔE is energy gap between I_523_ and I_524_, K_B_ is Boltzmann constant, and the T is absolute temperature. The temperature-dependent FIR (I_523_/I_558_) was shown in Figure 6b. In accordance with the FIR technique, FIR can be fitted well with a linear function which exhibits high fitting degrees (R^2^ ≥ 0.99).

Besides, the sensitivity of sensors plays a vital role in temperature measurement. According to the definitions, the relative sensitivity Sr can be expressed by ref. [29]. As an important flatness for evaluating the performance of temperature sensors, The formula for Sr as Equation (1).

(2) $$\mathrm{Sr}=|\frac{1}{\mathrm{FIR}}\frac{\mathrm{dFIR}}{\mathrm{dT}}|\;=\frac{∆E}{{\mathrm{KT}}^{\;2}}$$

According to Formula (2), the temperature measurement method based on the I_524_/I_559_ fluorescence intensity ratio at 310 K in the 310–670 K temperature range yields the maximum Sr. The Sr is maximum of 0.0028 K^−1^ (310 K). Presentation based on Figure 6d CIE1931, it is very straightforward to see that from 310 K to 670 K Er^3+^ are mainly concentrated in green light. The maximum sensitivity and the temperature measurement range of Yb^3+^/Er^3+^ in various hosts are shown in Table 1.

## 4. Conclusions

In summary, a low-temperature hydrothermal method was successfully developed for the synthesis of phase-pure orthorhombic Sc_2_Mo_3_O_12_ crystals. The effects of precursor molar ratio, reaction temperature, reaction time, and surfactant on the phase formation and crystal morphology were systematically investigated, leading to the successful preparation of phase-pure Sc_2_Mo_3_O_12_ crystals. Using phase-pure Sc_2_Mo_3_O_12_ as the host, efficient multicolor UCL was realized under 980 nm excitation by employing Yb^3+^ as the sensitizer and Er^3+^, Tm^3+^, or Ho^3+^ as the activator ions. More importantly, the phase-pure Sc_2_Mo_3_O_12_ host exhibited thermally enhanced UCL over the temperature range of 310–670 K without obvious thermal quenching. Furthermore, optical thermometric performance based on the FIR technique was demonstrated, yielding a maximum Sr of 0.0028 K^−1^ at 310 K. These results demonstrate that low-temperature synthesized Sc_2_Mo_3_O_12_ is a promising NTE host for thermally enhanced UCL and high-temperature optical thermometry, providing a feasible strategy for the low-temperature synthesis of NTE upconversion host materials and offering new insights into overcoming thermal quenching in high-temperature luminescent applications.

## Figures and Tables

**Figure 1:**
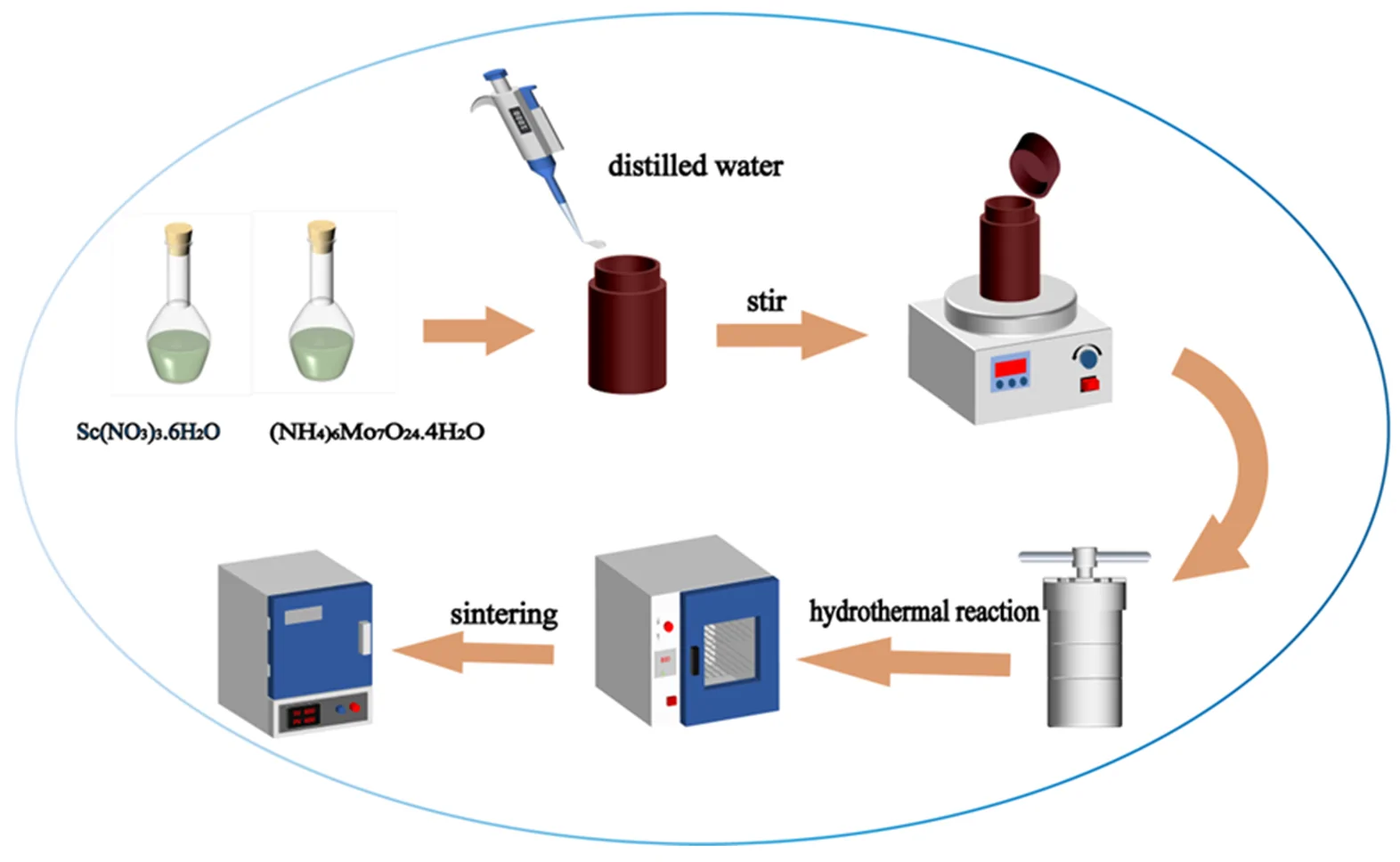
Schematic preparation of Sc_2_Mo_3_O_12_ using hydrothermal reaction.

**Figure 2:**
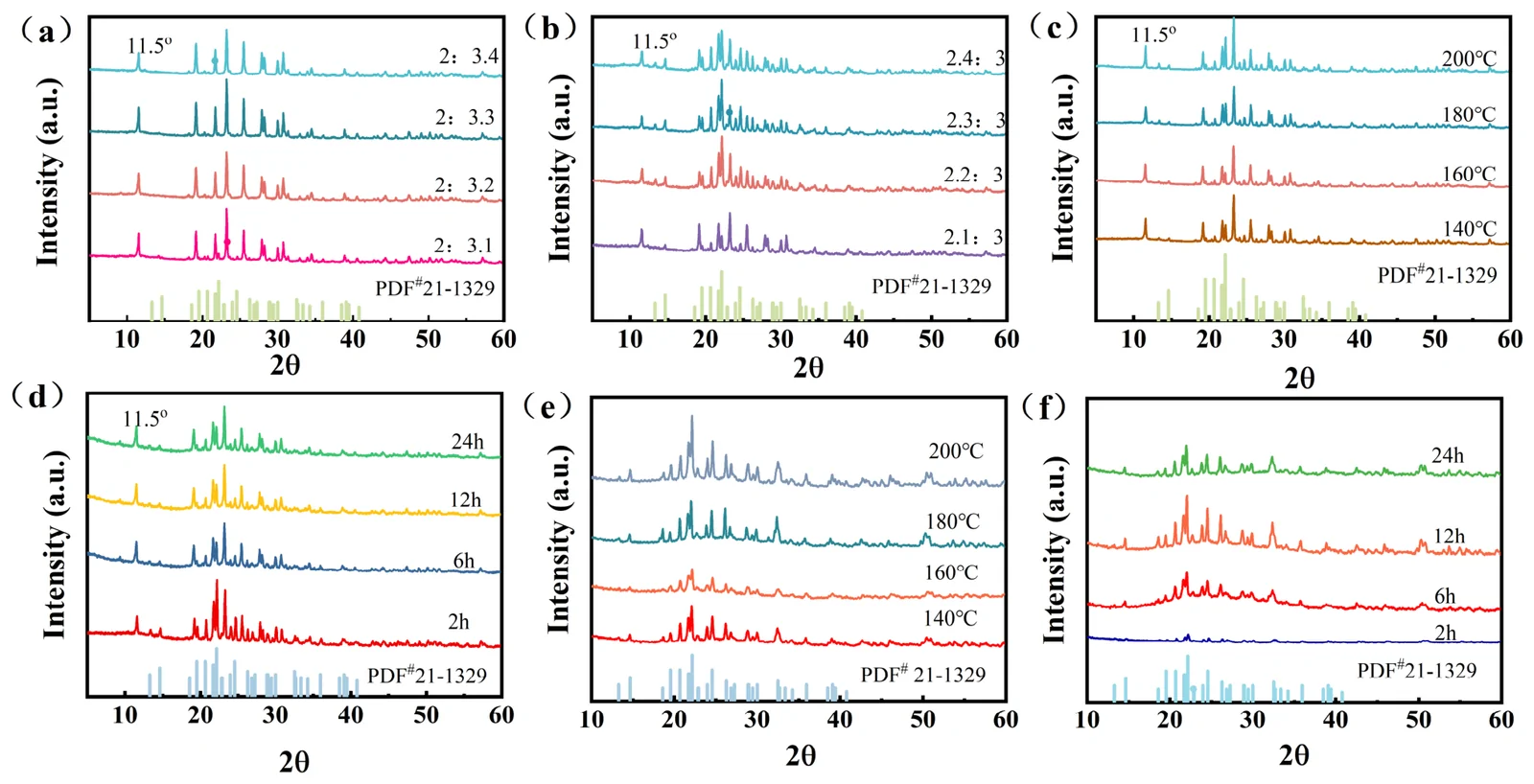
XRD patterns of Sc_2_Mo_3_O_12_ samples synthesized by hydrothermal reaction under different conditions. (**a**) at 140 °C for 2 h with different mole ratios (**b**) at 140 °C for 2 h with different mole ratios (**c**) at 2 h for various temperatures (**d**) at 140 °C for different times (**e**) at various temperatures for 2 h (**f**) at various time for 140 °C.

**Figure 3:**
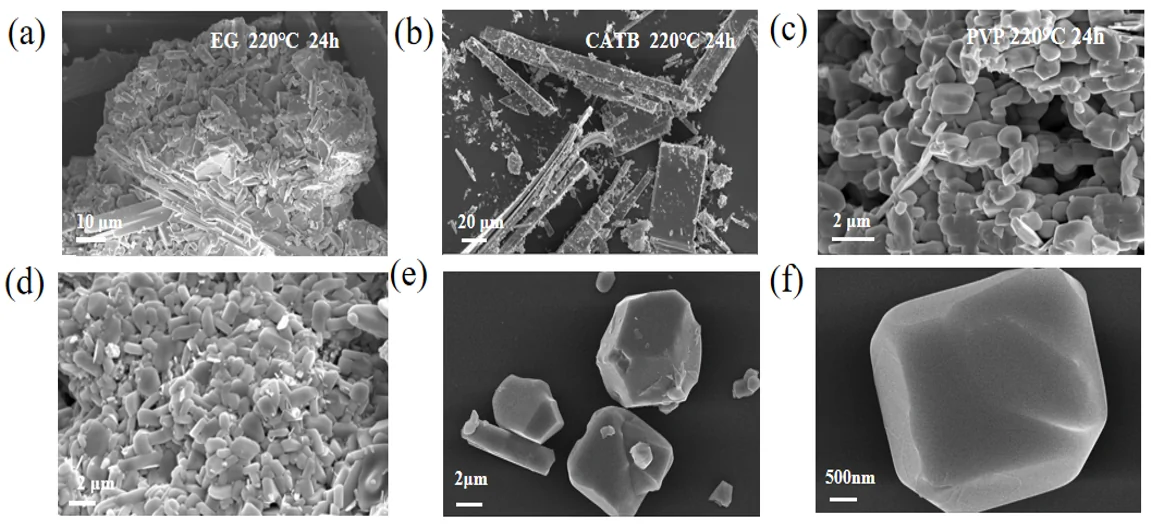
SEM images of Sc_2_Mo_3_O_12_ samples synthesized by hydrothermal reaction under different surfactants (**a**) at 220 °C for 24 h with 0.25 g ethylene glycol (**b**) with 0.25 g CATB (**c**) with 0.25 g PVP (**d**–**f**) microscopic morphology using PVP modification.

**Figure 4:**
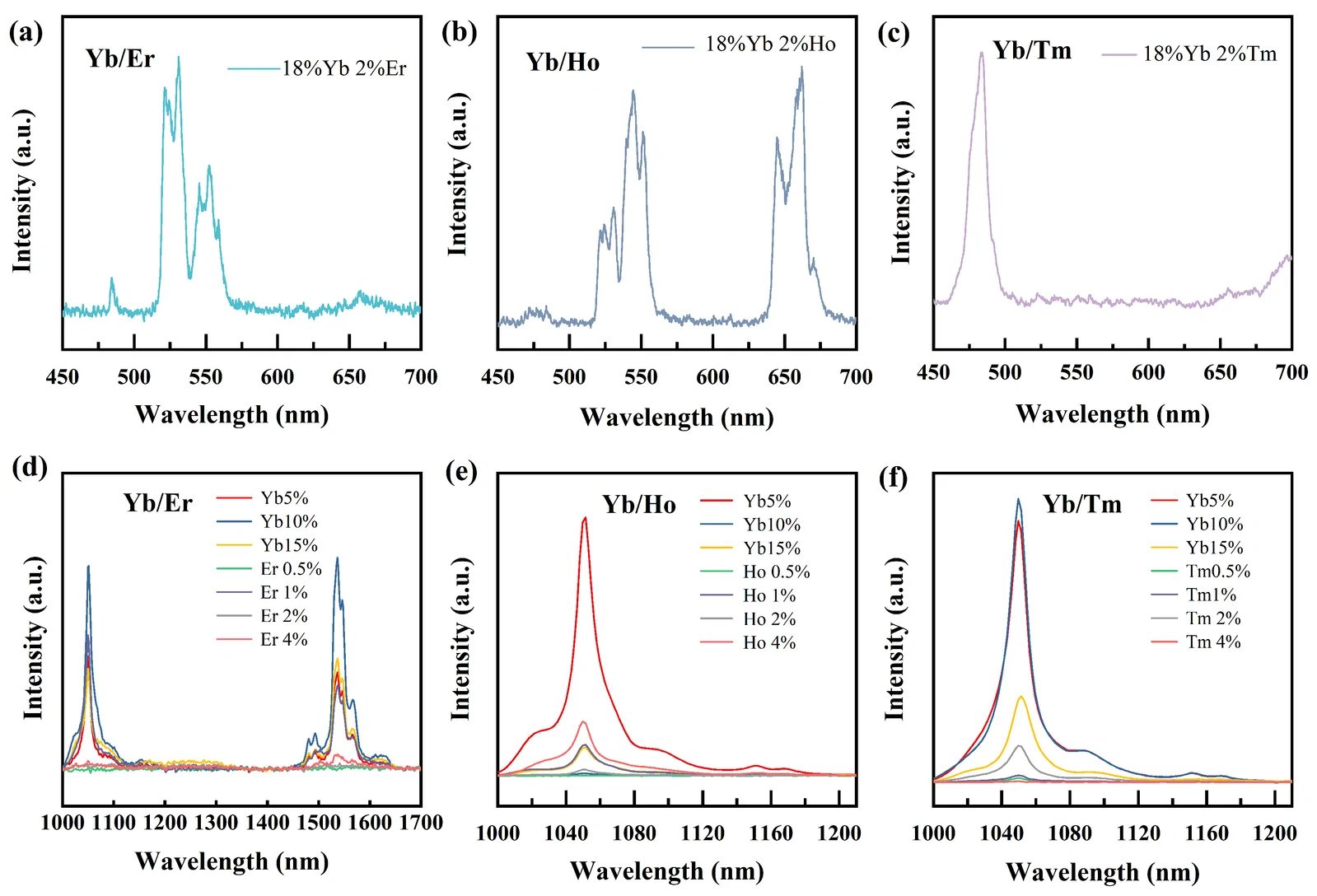
Upconversion emission spectra of Sc_2_Mo_3_O_12_ doped with (**a**) Yb/Er, (**b**) Yb/Tm, (**c**) Yb/Ho. Downconversion emission spectra of Sc_2_Mo_3_O_12_ doped with different molar ratios of (**d**) Yb/Er (**e**) Yb/Tm, (**f**) Yb/Ho.

**Figure 5:**
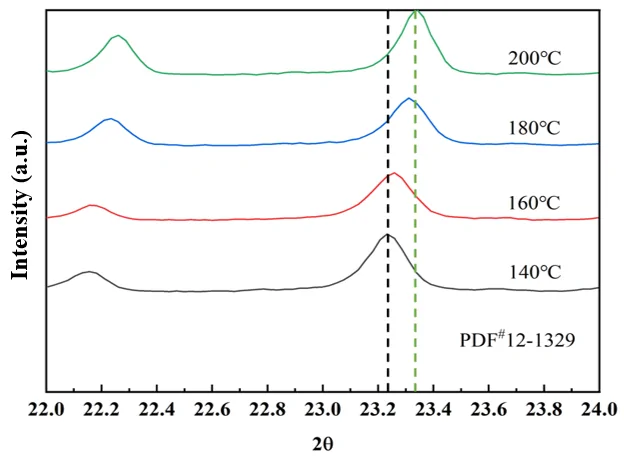
The high-resolution XRD patterns of the sample at different temperatures.

**Figure 6:**
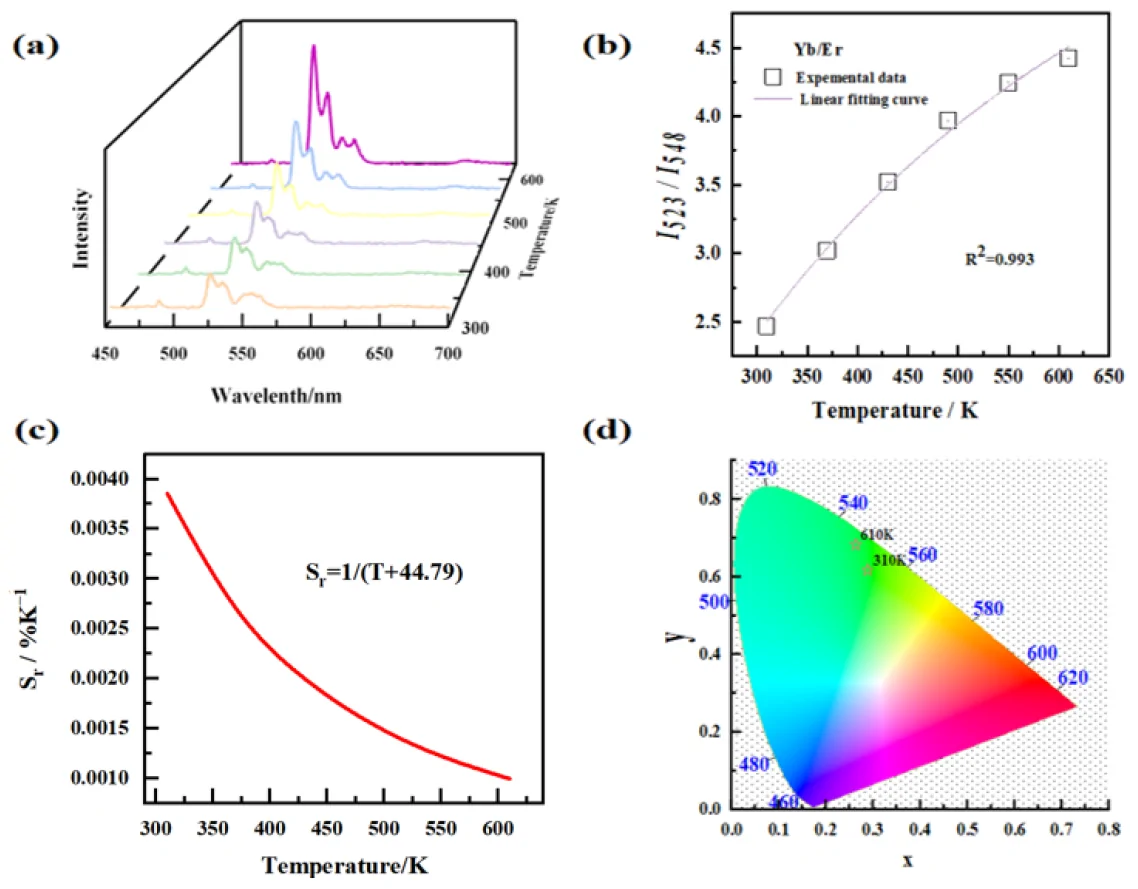
(**a**) UC emission spectra of Sc_2_Mo_3_O_12_: Yb/Er crystals at various temperatures The FIR (I_523_/I_548_) (**b**) as a function of temperature and (**c**) relative sensitivity (**d**) Commission International de I’Eclairage (CIE) chromaticity coordinates of optical emission from the Sc_2_Mo_3_O_12_:Yb/Er microcrystals with a different temperature.

**Table 1:** Comparative analysis of temperature sensing properties of lanthanide-doped optical materials.

Materials	λ_ex_ (nm)	λ_em_ (nm)	Temperature Range(K)	MaxS_r_ (%K^−1^)	Reference
NaYbF_4_:Er@NaYbF_4_:Tm	980	520,540	300–405	1.2	[30]
CaTiO_3_:Nd/Yb/Er	808	524,662	303–573	0.17	[31]
Y_2_O_2_S:Er/Yb/Tm	980	553,797	303–573	0.080	[32]
LuYO_3_:Er/Yb	980	523,553	298–848	0.0167	[33]
Gd2O3:Yb/Er	980	522,562	252–413	0.022	[34]
K_3_LuF_6_:Er,Yb	980	546,662	79–579	1.31	[35]
Sc_2_Mo_3_O_12_:Yb/Er	980	523,548	310–670	0.003	This work

**Figure A1:**
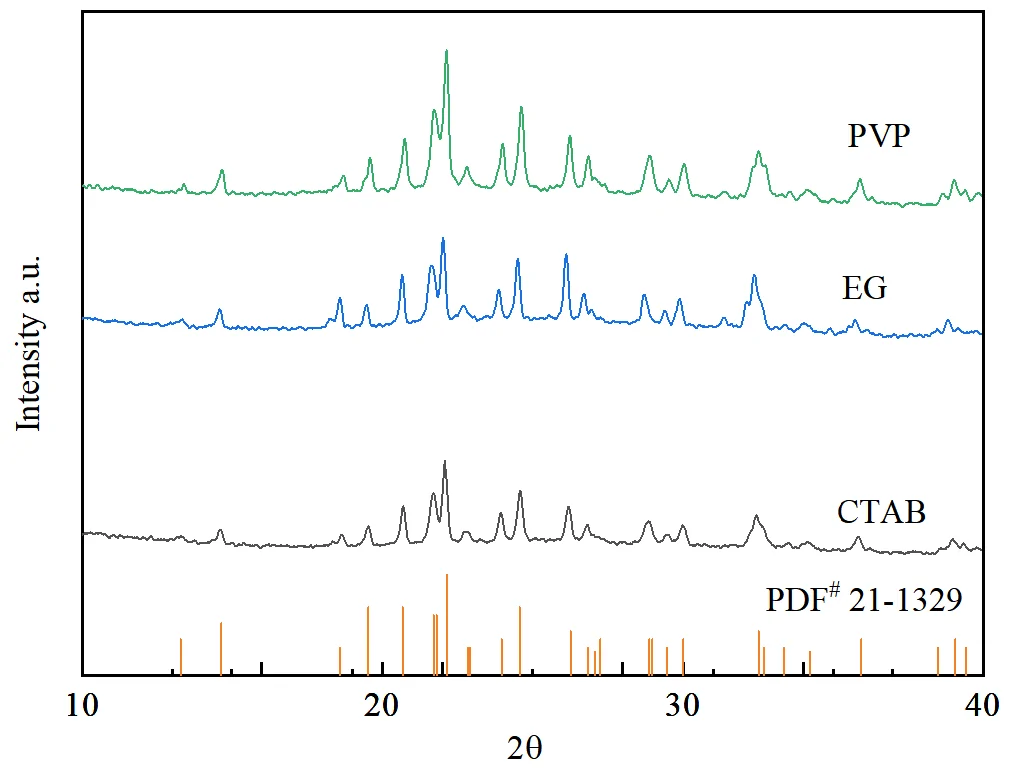
The XRD patterns of Sc_2_Mo_3_O_12_ materials modified with PVP, EG and CTAB.

## Data Availability

The authors confirm that the data supporting the findings of this study are available within the article.
